# Exosome Biogenesis and Biological Function in Response to Viral Infections

**DOI:** 10.2174/1874357901812010134

**Published:** 2018-09-28

**Authors:** Brennetta J. Crenshaw, Linlin Gu, Brian Sims, Qiana L. Matthews

**Affiliations:** 1 Department of Biological Sciences, Microbiology Program, College of Science, Technology, Engineering and Mathematics, Alabama State University, Montgomery, AL, USA; 2 Department of Medicine, Division of Pulmonary, Allergy & Critical Care Medicine, University of Alabama at Birmingham, Birmingham, AL, USA; 3 Departments of Pediatrics and Cell, Developmental and Integrative Biology, Division of Neonatology, University of Alabama at Birmingham, AL, USA

**Keywords:** Exosome, Biogenesis, Viral infection, Extracellular vesicles, RNAs, DNA

## Abstract

**Introduction:**

Exosomes are extracellular vesicles that originate as intraluminal vesicles during the process of multivescular body formation. Exosomes mediate intercellular transfer of functional proteins, lipids, and RNAs. The investigation into the formation and role of exosomes in viral infections is still being elucidated. Exosomes and several viruses share similar structural and molecular characteristics.

**Explanation:**

It has been documented that viral hijacking exploits the exosomal pathway and mimics cellular protein trafficking. Exosomes released from virus-infected cells contain a variety of viral and host cellular factors that are able to modify recipient host cell responses. Recent studies have demonstrated that exosomes are crucial components in the pathogenesis of virus infection. Exosomes also allow the host to produce effective immunity against pathogens by activating antiviral mechanisms and transporting antiviral factors between adjacent cells.

**Conclusion:**

Given the ever-growing roles and importance of exosomes in both host and pathogen response, this review will address the impact role of exosome biogenesis and composition after DNA, RNA virus, on Retrovirus infections. This review also will also address how exosomes can be used as therapeutic agents as well as a vaccine vehicles.

## 1 INTRODUCTION

Exosomes are minute (30-150nm) extracellular vesicles (EVs) that are generated during the maturation of endosomes [1]. Exosomes are secreted into the extracellular environment by several cell types, such as tumor and immune cells [2], antigen-presenting cells [3], and epithelial cells [4]. Exosomes are found in biological fluids such as blood [5], urine [6, 7], semen [6, 8], saliva [6, 9], cerebrospinal fluid [6, 10] and breast milk [6, 11]. Recently, exosomes have developed as an essential tool for facilitating intercellular communication through the transfer of biologically active RNAs, lipids, and proteins [1, 12].

Exosomes were first observed in the early 1980s by two groups studying the culture media of reticulocytes [13-15]. They revealed that small vesicles developed *via *inward budding inside of an intracellular endosome [13-15]. In the late 80s, the word “exosomes” was coined by Dr. Rose Johnstone [16, 17]. She observed that developing reticulocytes are composed of large sacs filled with miniature membrane-enclosed vesicles that were 30-100 nm [16, 17]. Later, she identified transferrin as a plentiful membrane protein on these sacs [16, 17]. Immunogold labeling that contained a monoclonal antibody alongside the transferrin receptor showed that the larger sacs joined with the cell’s plasma membrane, secreting the smaller membrane-enclosed structures [16, 17].

Since their discovery, the biogenesis, secretion, and composition of exosomes have been extensively studied [18]. When discovered more than 30 years ago, exosomes were originally thought to be a mechanism of discarding plasma membrane proteins in maturing reticulocytes found in red blood cells [13, 18]. These small membranous vesicles are generated by the inward budding of the plasma membrane to form intracellular endosomes [19]. Early endosomes join with endocytic vesicles and fuse their content with those intended for exocytosis, degradation, or recycling [16]. As the early endosomes mature, they develop into late endosomes and become characterized by the formation of Intraluminal Vesicles (ILV) or Multivesicular Bodies (MVBs) inside the lumen of the endosome [19]. The MVBs fuse with the plasma membrane, as well as lysosomes for degradation of their contents, releasing their contents into the extracellular environment in the form of exosomes [19] (Table **1**).

The processes that control the formation of ILVs inside MVBs and the fusion with the plasma membrane to release exosomes into the extracellular environment are not completely understood [18]. The biogenesis and secretion of exosomes are believed to be facilitated through the Endosomal Sorting Complex Required for Transport (ESCRT)-dependent pathway [13, 18] or ceramide-dependent pathway [18, 20]. The ESCRT machinery is composed of four components: ESCRT-0, I, II, and III [18, 21]. In conjunction with a variety of proteins, the ESCRT machinery is primarily involved in the binding, sorting, and clustering of ubiquitinylated receptors and proteins in the late endosome [18]. In the ESCRT-dependent pathway, components of the ESCRT machinery are consecutively transported to the endosomal membrane [18]. Along with transportation, they begin with the hepatocyte growth factor-regulated lyrosine kinase substrate (Hrs) and bind to the ubiquitinate protein ESCRT-0, the phosphatidylinositol-3-phosphate (PI(3)P), and 3,5-bisphosphate (PI(3,5)P2) through lipid-bind domains: GRAM-Like Ubiquitin-binding in EAP45 (GLUE) [18, 22] and Fab-1, YGL023, Vps27, and EEA1 (FYVE) [18, 23]. ESCRT-I and -II facilitate the budding of ILVs, in which cargo is transported into the lumen [18]. ESCRT-III is recruited by ALG-2 interacting protein X (Alix) to facilitate pulling, spiral formation, and complete budding [18]. Deubiquitinatings enzyme (DUB) deubiquitinate the protein and vacuolar protein sorting proteins (Vps) 4 reprocess the ESCRT machinery [18]. Next, the MVB is transported to the plasma membrane [18]. Through fusion, the ILVs are released into the extracellular environment and are denoted as “exosomes” [18].

In addition to the ESCRT-dependent pathway, current research has reported the presence of an alternate pathway referred to as the ceramide-dependent pathway [18, 20, 24]. The ceramide-dependent pathway is based on the development of glycolipoprotein microdomains (lipid rafts) in which sphingomyelin is transformed into ceramide by sphingomyelinases (enzymes generating ceramide from sphingomyelin) [18, 20, 24]. Ceramide accumulation then prompts microdomain amalgamation and initiates ILV formation within MVBs [18].

Exosomes are secreted in many cell types during normal, physiological, and pathological conditions [6, 25, 26]. The regulatory molecules involving the release of exosomes were reported by Ostrowski *et al.* [27, 28]. They reported that Rab27a and Rab27b were affiliated with exosome secretion [27, 28]. They also reported that knockdown of Rab27 and/or their effectors, EXPH5, and SYTL4, could prevent the secretion of exosomes in HeLa cells [27, 28]. Another study was reported by Baietti *et al.* [27, 29]. They reported that syndecan-syntenin networked with ALIX protein through Leu-Tyr-Pro-X(n)-Leu motif to support the intraluminal budding of endosomal membranes [27, 29]. Moreover, Yu and colleagues revealed that the tumor repressor protein p53 and its downstream effector TSAP6 could augment exosome production [27, 30]. Overall, these findings suggest that molecules are responsible for the formation and secretion of exosomes in parent cells; and can act as a regulatory network [27].

Exosomes have been purified and isolated from *in vitro* cell cultures and biological fluids using several techniques [31]. The most common method used to isolate exosomes from cell culture supernatants involves a series of ultracentrifugation to eliminate cells and excessive debris [31]. A series of ultra-centrifugation is performed to pellet the exosomes. The pellet is washed in a vast amount of phosphate buffer saline (PBS) to remove contaminating proteins. The pellet is then centrifuged an additional time at an equivalent high speed. The size of exosomes is equal to that of the ILVs of the MVBs from where they are derived [3]. Additional isolation can be accomplished by polymer-based precipitation [32], immuno-affinity capture [33], size exclusion chromatography [34], microfluidics [35], and ultrafiltration [34]. Due to the rising interest in exosomes for therapeutics, commercially available kits (Exosome Isolation kit, Exoquick, and Exo-spin) [3] are available to isolate exosomes.

The assessment of the purity of exosomal preparations, in addition to their composition, is often difficult based on the cell type/origin from which the exosomes are derived [5]. Furthermore, it is known that there is a relationship between exosomal trafficking and viral hijacking [36]. Therefore, when isolating exosomes from virus-infected cells, it is imperative to ensure that the collected material only contains exosomes and no viral particles. Konadu *et al*. Optimized many exosome characterization techniques [1]. These techniques included using electron microscopy to visualize exosomes, examining exosomal protein markers (Annexin V, CD63, CD81, *etc*.) through immunoblot analysis, and performing nanoparticle tracking (NanoSight) analysis to measure and analyze the size of exosomes [1, 37]. Because of their tiny size, exosomes cannot be identified by using standard flow cytometry. Nonetheless, the existence of epitopes on the surface of exosomes can be used to sort/purify exosomes [38].

Recent studies have acknowledged exosomes as vital components in viral pathogenesis and immunity [1]. Exosomes allow the host to mount effective immune responses against pathogens, which includes activating antiviral mechanisms and transferring antiviral elements among a variety of cells [39]. Depending on the nature of the target and pathogen, exosomes can increase or restrict an infection [1]. Exosomes that are composed of viral genomes can stimulate viral spread by entering susceptible cells while eluding immune recognition [40]. In certain cases, exosomes that contain nucleic acids or viral proteins initiate immune responses in myeloid cells [41]. Research on Dengue Virus, human T-cell lymphotropic virus (HTLV), Hepatitis C Virus (HCV), and Human Immunodeficiency Virus (HIV) has proved that exosomes distributed from infected cells transport various regulatory factors, (as reviewed in (Chahar 2015)). These factors include host functional genetic elements to neighboring cells, cellular and viral miRNA, proteins, and viral RNA which help aid in regulating cellular responses and producing infections [1]. This review will summarize the biogenesis of exosomes and their role in biological functions in response to viral infections.

## 2 HIJACKING BY VIRUSES EXPLOITS THE EXOSOMAL PATHWAY

Viruses take control of and exploit cellular replication mechanisms to replicate. When successful, most viruses end up killing their host cell. Some viruses can hijack affiliates of vesicular trafficking and through a sequence of complexes known as ESCRT. Also, they can assimilate viral constituents into exosomes. Viral antigens in exosomes maximize persistence by hiding viral genomes, entrapping the immune system, and maximizing viral infection in uncontaminated cells. Exosomes can be used to present a source of viral antigens that can be targeted for therapeutic uses. Also, exosomes can be used as a biomarker for disease in the regulation and obliteration of some illnesses [42].

Several viruses enter cells through the endocytic pathway. Viruses that enter through endocytosis can hijack and use exosomal pathways for their own benefit. Infectious diseases, such as HCV, Zika virus (ZV), West Nile virus (WNV), and DENV enter this pathway by clathrin-mediated or receptor-mediated endocytosis [43-46]. After “back-fusion” of ILVs, these infectious viruses antagonize late endosomes, thus resulting in the discarding of the viral genome inside of the cytoplasm [44]. In the case of HCV, the viral genome can linger in ILVs and be secreted inside of the exosomes, where they can perform as infectious particles [41-43]. The mobility of vesicles in the endosomal pathway as they maneuver through the plasma membrane offers numerous chances for a viral disturbance. Viruses can penetrate the endosomal pathway by entry and viral fusion inside of the cell.

Current studies have shown that hepatocyte-derived exosomes that contain HCV RNA can stimulate innate immune cells [43]. HCV can infect specific target cells in the form of cell-free viruses and through cellular contact [47]. In 2013, Ramakrishnaiah *et al*. reported that HCV infection can be spread by exosomes between hepatocyte-like cells and can create a productive infection [43]. Another study was reported by Dreux and colleagues [48]. They demonstrated that hepatocyte-derived exosomes containing viral RNA can prompt IFN-α production in plasmacytoid dendritic cells [48]. In 2014, Liu *et al*. reported the presence of HCV in exosome-free and exosome-related forms [47]. The group revealed that exosome-associated HCV was infectious and resistant to neutralization by an anti-HCV neutralizing antibody [47]. In addition, they revealed that more exosome-associated HCV was present compared to exosome-free HCV identified in the plasma of HCV-infected patients [47]. Therefore, this evidence suggests that exosome-associated HCV serves as an alternate form for HCV transmission and infection [47].

## 3 EXOSOMES AND PROTEIN TRAFFICKING

Several viruses express membrane proteins [49]. Membrane proteins in enveloped viruses are often structural constituents of the virus that facilitate the important task of membrane fusion and receptor recognition. The activities of these proteins entail that they are copied properly in infected cells. Often, these cataloging events rely on the ability of the virus to make a network with the cellular trafficking machinery and copy cellular protein trafficking signals. Notably, modification or loss of these signals can impact viral pathogenesis and infectivity [50].

Exosomes found in body fluids play an essential role in exchanging information among cells. There are three mechanisms of contact between exosomes and their recipient cells. First, the transmembrane proteins of exosomes make a network directly with the signaling receptors of target cells [51]. Secondly, the exosomes join with the plasma membrane of recipient cells and transport their content inside of the cytosol [52]. Third, the exosomes are incorporated into the recipient cells where they have two possible fates. In the first possible fate, engulfed exosomes may join inside of the endosomes and go through transcytosis. This process allows the exosomes to move across the recipient cells and be released into neighboring cells. If the second possible fate occurs, endosomes can fuse with engulfed exosomes and undergo degradation within the lysosomes [52, 53]. Studies have recently shown the factors that have influenced the internalization of exosomes in recipient cells. Koumangoye *et al*. detected that disruption of exosomal lipid rafts leads to the inhibition of internalization of exosomes [27, 54]. The group also showed that annexins were necessary for the uptake of exosomes in the breast carcinoma cell line BT-549 [27, 54]. Escrevente *et al*. described the importance of protease K and exosome-mediated uptake of exosomes derived from the human ovarian carcinoma cell line SKOV3 [27, 55].

Exosome trafficking allows tetraspanins and a variety of cellular constituents, such as major histocompatibility complex class II (MHC-II) molecules to maneuver through the cell. The process of “back fusion” can aid in the entry of viral proteins that are being generated inside the cell and targeted for the endosomal compartment [44]. Viruses, such as herpesviruses and other DNA viruses that require proximity to the nucleus and endoplasmic reticulum, will also follow this route and possibly have components incorporated into the exosomes [42] (Table **1**).

## 4 EXOSOMES AND VIRAL INFECTIONS

Exosomes have several characteristics that are like some viruses. These characteristics include biogenesis, molecular properties uptake by cells, and exosome-mediated intercellular transfer of functional RNAs, mRNAs, and cellular proteins [12]. The differences between exosomes and some viruses include self-replication after infection of new cells, temporary regulated viral expression, and the complexity of viral entry [56]. Several virus-infected cells secrete exosomes that vary from their virion counterparts but may consist of numerous RNAs and viral proteins. To some degree, exosomes have been evaluated for their content and attributes during viral infection [12]. However, more investigation is needed, as it relates to DNA viruses. Table **1** summarizes the relationship of DNA and RNA virus infection and exosomes.

## 5 RETROVIRUSES

Retroviruses are enveloped RNA viruses that bud at the plasma membrane of infected cells [57]. They utilize reverse transcriptase to change their single-stranded RNA into double-stranded DNA. This allows the reverse transcriptase to become integrated inside the genome of the cells which have been infected [58]. All retroviruses are comprised of three key coding domains i) ***gag***, ii) ***pol***, and iii) ***env*** [59]. ***Gag*** guides the synthesis of internal virion proteins that make up the nucleoprotein structures, the matrix, and the capsid. ***Pol*** comprises the information that is needed for integrase and reverse transcriptase and enzymes. ***Env*** descends the transmembrane and surface constituents of the viral envelope protein. ***Pro*** is a smaller coding domain. It encodes the virion protease in all retroviruses. In most cases, simple retroviruses only carry this primary information. However, complex retroviruses code for non-virion proteins that develop from various spliced messages [59].

Retroviruses are “retro” because they reverse the route of the standard gene copying procedures [60]. According to the Trojan exosome hypothesis, retroviruses could be exosomes that developed upon the mutation of a structural *gag* gene [61]. The *gag* gene is encoded by an integrated retrotransposon that directs viral biosynthesis to the exosome pathway [61]. The most studied retrovirus that infects humans is HIV-1 [62].

## 6 HUMAN IMMUNODEFICIENCY VIRUS

The potential functions of exosomes secreted during HIV-1 and HIV-2 infections are just beginning to be explored. HIV-1 and HIV-2 share the same modes of transmission [63]. Immunodeficiency develops gradually in people that are infected with HIV-2. HIV-2 infected people are less infectious in the early phase of infection as compared to those infected with HIV-1. As the disease progresses, HIV-2 increases. However, in the case of HIV-1, as the disease progresses, HIV-1 decreases [63, 64].

HIV-1 and HIV-2 are two distinct viruses that infect monocytes/macrophages and T lymphocytes. These types of cells express CD4, a cell surface type I transmembrane glycoprotein, which is the major receptor for HIV [65]. Host cell infection starts with the involvement of the gp120 subunit of the viral envelope (Env) glycoprotein-network through CD4. This involvement leads to variations in gp120 that increase its affinity to chemokine co-receptors, CXCR4 or CCR5. Co-receptor binding to Env initiates changes in the gp41 subunit. These changes stimulate fusion and tethering of the Env to the cell plasma membrane [66, 67]. CD4-Env collaboration is essential for viral entry. Therefore, the use of participant particles that target this interaction [65] or the downregulation of CD4 surface expression [68] can inhibit HIV-1 infection.

Exosomes display similar molecular and structural characteristics with HIV-1 and HIV-2. They are both enclosed by a lipid bilayer. Their size and density [69] vary from 50 to 150 nm in diameter [70] and 1.13 to 1.21 g/mL [71], correspondingly. Also, they are both composed of RNA species [61], carbohydrates [72], lipids [73], and proteins [14, 74]. Exosomes derived from HIV-infected cells are enhanced with viral RNAs [75, 76] and Nef protein [77].

Because of these comparisons, studies have shown that HIV-1 can be produced by the identical pathways of exosome biogenesis [39, 61]. In favor of the Trojan exosome hypothesis, HIV-1 recruits constituents of the host ESCRT machinery to the appropriate sight of viral budding [12, 39]. The collaboration between tetraspanin and HIV-1 Gag protein proposes that HIV-1 may use lipid raft micro-domains rich in tetraspanins for virus assembly [78]. Tetraspanins CD63 and CD81 are located on the surface of some exosomes. CD63 and CD81 are known to be involved with HIV-1 infectivity [79]. In addition, exosomes and HIV-1 express sialyllactose-containing gangliosides. Studies have shown that sialic-acid-binding immunoglobulin-like lectins (Siglecs)-1 interact with sialyllactose-containing ganglioside on exosomes and HIV-1. Siglecs-1 stimulates mature dendritic cell (mDC) capture and storage of both exosomes and HIV-1 in mDCs. This facilitates trans infection of T cells by mDCs [80].

Studies have shown that exosomes that are released from infected cells contain co-receptors for HIV-1 which can improve virus entry inside cells [81]. Expression of the viral Nef protein modifies the endosomal network by maximizing the amount of MVBs, endosomes, and lysosomes. Nef is a protein that is coded by HIV-1 and HIV-2 genomes. The Nef protein modulates protein trafficking and signal transduction mechanisms in infected cells (39). Recent studies have shown that Nef is displayed in exosomes that are derived from cells. Studies have also shown that Nef-containing exosomes prompted apoptosis in CD4+ T cells. Thus, exosomal Nef can participate in HIV-1 and HIV-2 pathogenesis by aiding in the reduction of CD4+ T cells [12].

Nef promotes MHC-I molecules and the downregulation of CD4’s cell surface. Nef attaches itself to the cytosolic tail of MHC-I and CD4+. Due to this attachment, it disturbs the intracellular trafficking of these proteins through reformed mechanisms [82]. Nef stimulates CD4 endocytosis *via *clathrin-coated vesicles. This allows the mechanism to form a tripartite complex in the clathrin pits with the adaptor protein (AP) complex 2 and CD4 [83] at the plasma membrane. In contrast , Nef inhibits MHC-I from attaining the plasma membrane by stimulating the retention of these molecules inside the Golgi apparatus by interacting with AP-1 [82]. Also, Nef can prompt MHC-I internalization by initiating a signal transduction pathway that involves the assembly of a kinase cascade [84]. Both replicas believe that Nef transmits MHC-I molecules toward MVBs and late endosomes inside the endolysosomal pathway. In addition, MHC-I and CD4 are directed to MVBs upon Nef expression [85].

## 7 EXOSOMES AUGMENT HIV-1 ENTRY

As previously mentioned, exosomes can play a vital role in the entry of HIV-1 viral infection. HIV-1 utilizes primary and secondary cellular receptors in order to invade host cells [86]. These receptors facilitate precise, high-affinity collaborations with viral entry proteins. In addition, these receptors prime the entry protein for consequent phases in the viral entry method [87]. HIV-1 has an Env that it secures when it leaves the cells. In order to infect cells, the HIV-1 Env protein fuses near the primary cellular receptor and then to the cellular co-receptor. Sequentially, this binding causes fusion between the host and viral cell membranes which aid in the initiation of infection [88].

HIV-1 uses human T-cell immunoglobin mucin (TIM) proteins to augment viral entry. TIMs are a group of proteins (TIM-1, TIM-3, and TIM-4) that promote phagocytosis of apoptotic cells [87]. The interaction of dying cells and enveloped viruses is mediated *via *phosphatidylserine (PtdSer). PtdSer is a phospholipid that acts as an assistant constituent for viral fusion, using [86, 89] residues that are exposed to the cellular and viral membranes [87]. In previous reports, Sims *et al*., showed that neural stem cell (NSC)-derived exosomes have TIM-4 protein. A few years later, the group showed that TIM-4 is involved in HIV-1 exosome-dependent cellular entry mechanism. Also, they showed that HIV-1, which consists of large quantities of PtdSer, could bind to PtdSer receptors on exosomes, such as TIM-4. Thus, they concluded that exosomes from multiple sources, such as breast milk, blood, NSC, and human epithelial lung cells (A549), increased HIV-1 cellular entry. Sims and the group also demonstrated that exosome-mediated entry was effectively blocked using a TIM-4 antibody [86].

**Fig. (1):**
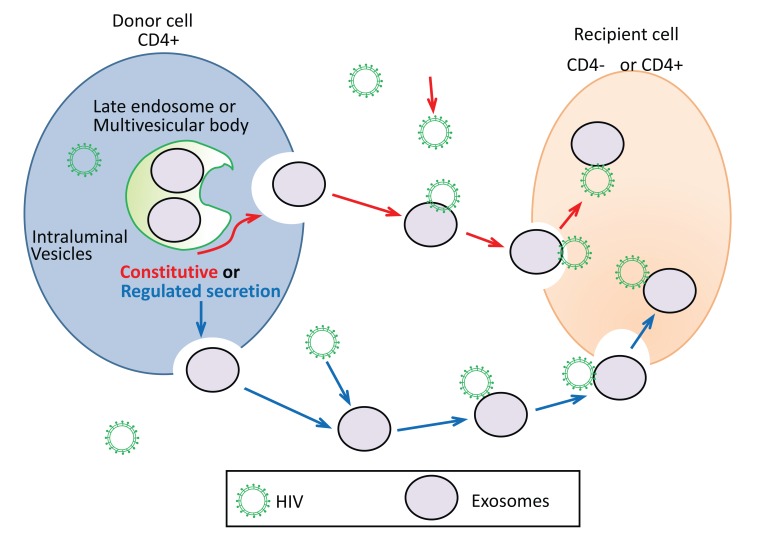
**
Proposed mechanisms of exosome secretion and HIV-1 interaction.** Exosomes can be secreted by means of a constitutive or regulated pathway. Exosomes can bind to HIV-1 *via *TIM-4, CD9, and/or CD81. This binding can facilitate exosome-mediated entry into CD4+ or CD4- cells. HIV-1 binds to exosomes released from the ‘donor’ cell and is shuttled to the ‘recipient’ cell.

Fig. (**1**) depicts the proposed mechanism of putative exosome-mediate HIV-1 entry in CD+4 or CD-4 recipient cells. As previously mentioned, the formation of exosomes occurs when the late endocytotic constituents join with the plasma membrane. This process can be either constitutive or regulated (Fig. **1**). Constitutive release of exosomes represent secretion of exosomes that is constant. Whereas, regulated exosome release represents exosome release that is the result of a stimulus or signal. Constitutive secretion of exosomes is seen in most types of cells, such as epithelial cells, EBV-transformed B cells, and immature dendritic cells [4, 90, 91]. Other cells can have regulated secretion of exosomes such as mast cells and T cells. Calcium has been shown to be a key regulator of this process [92, 93]. Activated CD4+ T cells were initially described to secrete EVs which were associated with FasL and APO2L (68). The secretion occurred in a putative mechanism to maintain immune tolerance and T cell homeostasis through the apoptosis of targeted cells [94]. HIV-1 infects CD4+ T cells and evades the host immune response. It also stimulates an inflammatory immune response which can cause cell exhaustion [95]. It has been well noted that HIV-1 enters T cells by binding and engagement of co-receptors and CD4. However, based on the findings of Sims *et al*. [86], it is speculated that HIV-1 can interact and bind secreted exosomes and enter into CD4+ and CD4- negative cells (Fig. **1**). Sims et. al proposed the mechanism of interaction of exosomes and HIV to be mediated by TIM-4, CD9, and/or CD81. It is possible that this interaction between exosomes and HIV-1 is specific to the origin of the exosome; therefore, further studies are required [96, 97].

## 8 DNA VIRUSES HIJACKING

Viruses are categorized according to the proteins that are encoded in the genome or viral genetic material, such as DNA [49]. DNA viruses are intracellular parasites that can only reproduce in cells. They can program the cell to reproduce the virus using the genes that are obtained inside the viral DNA genome. DNA viruses replicate their genomes with DNA polymerases and RNA polymerases [98]. With respect to the viral replication cycle, many DNA viruses control gene expression in a “time-ordered” approach. The virus expresses “early genes ” and “late genes.” The early genes interact with the cell to initiate the cell's DNA machinery. The late genes are mostly virion proteins that are essential for virion assembly. Except for poxviruses, all DNA viruses reproduce inside the nucleus of the infected cells. These viruses are dependent on a lesser or greater amount of the cell's DNA machinery. Most cells in the host are in a latent state, in which DNA synthesis is not accessible for viral DNA replication [99]. The extracellular form of a virus is called a virion. The virion is composed of a group of DNA genes that are sheltered by a protein containing coat termed as capsid. Symmetry and regularity categorize the capsid’s structure. It can invade and bind to cells. In some DNA viruses, the capsid is covered by a membrane, which is made from cellular membranes [49].

One DNA virus, called adenovirus (Ad), binds the cell surface Coxsackie virus and Adenovirus Receptor (CAR) on host cells, resulting in the entry of the virion into the host cell [36]. Entry of Ads inside the host cell is started by the knob domain of the fiber protein that binds to the host receptor. Next, a specified motif in the penton base protein makes a network with αv integrin. This stimulates internalization of the adenovirus *via *clathrin-coated pits, which can result in the entry of the virion inside the host in an endosome. Following internalization, the endosome acidifies which modifies the topology of viruses and causes capsid constituents to separate. As a result, the virion is released into the cytoplasm. The virus is then delivered to the nuclear pore complex where viral gene expression can occur (National Institutes of Health 2002).

For gene therapy approaches, adenovirus serotype 5 (Ad5) has gained increasing attention due to their ability to infect different types of cells. However, natural Ad tropism doesn’t allow Ad to infect cells that are deficient for CAR. In this regard, Sims *et al*. investigated the role of NSC-derived exosomes to traffic Ad into a CAR-deficient cell line, mouse B cell line A20 [100]. This group showed that NSC-derived exosomes facilitated substantial cellular entry of Ad5 in a receptor-independent manner. They showed that TIM-4 found on primary mouse NSC-derived exosomes play an essential role in the cellular entry of Ads. In addition, the group showed that treatment with TIM-4 antibody significantly blocked the exosome-mediated Ad entry [86, 100].

**Fig. (2):**
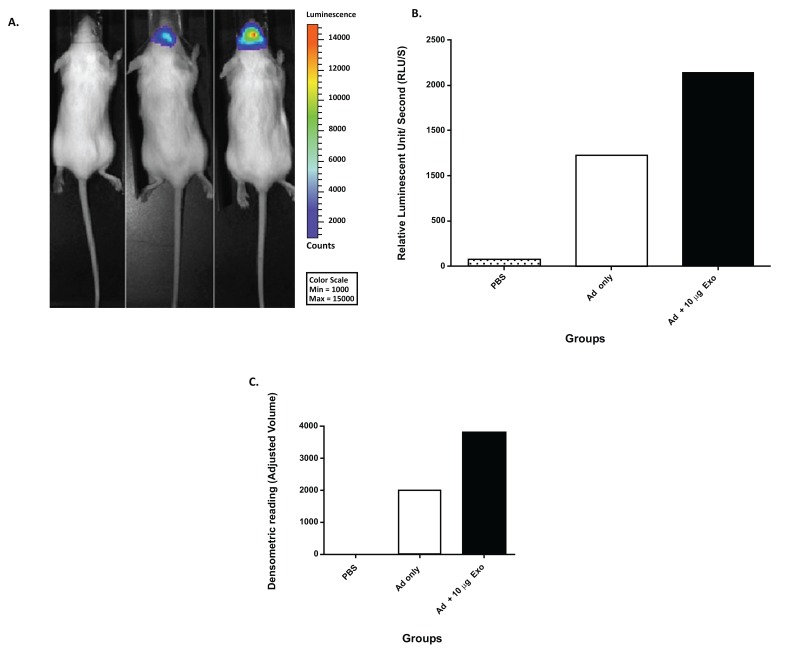
**Mouse NSC-derived exosome-mediated *in vivo* enhancement of Ad infection ** (unpublished work by Matthews *et al*.). **(A)** Mice were injected with PBS, 1x10^8^ Viral Particles (VP) of Ad, or 1x10^8^ VP of Ad co-incubated with 10 µg of exosomes. At 24 hours, post-injection mice were imaged by means of non-invasive luciferase imaging. The imaging results were represented as total luminescent counts. **(B)** Quantitation of representative mice from Fig. (**2A**), data represented as relative luminescent counts per second. **(C)** Densitometric analysis of western blot analysis of mouse brains at 48 hours post-injection (PBS, 1x10^8^ VP of Ad, or 1x10^8^ VP of Ad co-incubated with 10 µg of exosomes). Anti-adenovirus antibody was used for western blot analysis.

Sims and colleagues hypothesized that exosome-mediated viral entry not only occurs *in vitro* but occurs *in vivo* also (unpublished work). To test their hypothesis, Sims *et al*. co-incubated NSC-derived exosomes with an Ad5 vector (expressing luciferase after infection) and evaluated the ability of exosomes to mediate viral entry. After co-incubation, the Ad5 and exosome complexes were administered intranasally to mice and compared to Ad5 only and PBS. The data illustrated luciferase activity in the mouse group injected with Ad5 only. In addition, there was an increase in luciferase activity in animals injected with Ad and co-incubated with 10 µg/ml of exosomes (Fig. **2A**-**B**). To further confirm these findings, the mouse brains were extracted, sliced, and analyzed by western blot analysis. The results of the western blot analysis were reported *via *densitometric means (Fig. **2C**). This data demonstrated that NSC-derived exosomes could transfer Ad to the brain in an Ad receptor-independent process *in vivo*. This work could have implications for gene therapy applications targeting the brain.

Innovative biological research has shown how some viruses hijack cells by copying a signaling indicator to eradicate the body’s resistance. The virus destroys a defensive protein by using cell signals to inhibit it. Viruses replicate themselves by hijacking cellular processes. To replicate, viruses must distribute their DNA inside the nucleus of a cell. This allows a viral infection to cause conflict between the foreign DNA of the virus and the DNA of the host cell.

As a way of penetrating the cell’s defenses, viruses initiate their attack by interacting with cellular proteins. Weitzman *et al*. showed that the viral protein ICP0 utilizes phosphorylation- a biochemical reaction that is used in the cells to stimulate exchanges among proteins and cell signaling response to DNA damage, in Herpes Simplex Virus-1 (HSV-1) [101]. In HSV-1 infection, the phosphorylation signal on ICP0 attracts a cellular DNA damage response protein called RNF8. RNF8 binds to the false signaling marker and is then destroyed. Since RNF8 prevents viral replication, its destruction leaves the cell prone to HSV-1 infection, allowing the virus to take over the cell’s machinery [101]. To survive and replicate, the virus binds to the host cell and inserts its RNA or DNA inside the host. The viral DNA takes control of the cell machinery and creates multiple copies of its genomic material thus resulting in several new virions.

## 9 IMMUNE RESPONSE TO DNA VIRUSES

The immune system defends itself against viral infections by innate and adaptive immune responses [102]. The innate immune system serves as the major line of defense against pathogens, which is also needed for preparing for adaptive immunity. Innate immune responses are started by pattern-recognition receptors (PRRs). PRRs identify Pathogen-Associated Molecular Patterns (PAMPs), molecular structures of pathogens. The infection of virus activates a sequence of signaling procedures that lead to proinflammatory cytokines and transcriptional induction of type I interferons (IFNs). Recently, studies have reported vital information pertaining to RNA virus-triggered signaling pathways and the mechanisms of viral RNA recognition. Conversely, it remains uncertain on how DNA virus infection is identified by host cells and how it initiates the host antiviral defense. There are ten potential viral DNA sensors that have been reported. However, they have not been confirmed as consistently used sensors for distinctive DNA viruses in different animal and cell types [102].

Immune responses towards DNA are not limited to IFN-inducing pathways. Cytosolic DNA initiates caspase-1-dependent development of pro-inflammatory cytokines interleukin (IL)-18 and IL-1β. This pathway is facilitated by a Pyrin- and HIN200-domain-containing (PYHIN) protein called AIM2. Current data from knockout studies have shown the significance of AIM2 in host defense to DNA viruses. IFI16 is one of the most recent PYHIN proteins that have been acknowledged. IFI16 plays an important role in viral DNA attachment and the immune response of certain DNA viruses. Like AIM2, IFI16 joins viral DNA through HIN domains. IFI16 activation prompts inflammatory cytokine assembly and IFN-β in response to cytosolically administered HSV-1 or viral DNA infection [103].

Major pathogenic DNA viruses, such as human papillomavirus, polyoma virus, HSV, and EBV, have used many tactics to evade and weaken the immune response [104]. Recent studies have shown that these viruses modify recipient host cell responses. This can lead to the formation of productive infections. In addition, many viruses obtain host genes. This is needed to transform them for a specific purpose to avoid targeting by the immune system. Controls of viral infections are often facilitated by the stimulation of innate and adaptive immune mechanisms that include several types of cells. B cells, CD4+ T cells, and CD8+ T cells contribute to anti-viral responses in an antigen-specific fashion. This contribution is done by antibody secretion, cytolytic effect, or IFN-γ production, to guarantee extended protection. Alternatively, the stromal cells, NK-cells, and antigen-presenting cells play a major role in virus control during the early phase of infection. Though type I IFNs are important in forming early anti-viral defenses, other cytokines, such as IL-1β, IL-18, and IL-12 contribute to early defense in anti-viral immunity [102].

## 10 ROLE OF EXOSOME BIOGENESIS IN IMMUNE RESPONSES TO VIRAL INFECTION

Host cell machinery utilizes vesicle secretion as a defense mechanism against viral infection [12]. Secreted vesicles may stimulate immune cells and present viral antigens during a cellular response. For instance, the apolipoprotein B mRNA editing enzyme catalytic subunit 3G (APOBEC3G),-a cytidine deaminase that participates in the antiviral cellular network against retroviruses, can be packed and delivered to neighboring cells through exosomes to prevent HIV-1 replication [12]. Results from Izquierdo Useros *et al*. showed that HIV-1 uses DCs as a transit location in the non-replicative stage [80]. The virus packages all viral antigens and particles in exosome-like vesicles after fusing with DCs [80]. Exosomes bearing viral antigens deliver their cargo to CD4+ T cells and provoke infection [40] (Table **1**).

Exosomes can be used as therapeutic agents to modulate immune responses [105]. Exosomes mediate intercellular communication *via *innate and adaptive immune responses [56]. A variety of cells (B and T lymphocytes and dendritic cells) of the immune system have been shown to release exosome vesicles displaying immune modulatory properties [56].

The immunological properties of exosomes were first reported in 1996 by Raposo and her collaborators [90]. The group reported that B lymphocytes infected with EBV released exosomes that contained MHC II molecules that were essential to adaptive immune response [90]. In addition, they reported that these exosomes could activate specific CD4+ T cell clones *in vitro*, suggesting that they could play a role in adaptive immune responses [90]. In another study, Pegtel *et al*. showed that infected EBV derived exosomes deliver miRNA cargo [76]. They showed that these exosomes could move through the bloodstream and affect healthy recipient cells [76].

Exosomes can influence major tumor-related pathways [106]. Exosomes can alter tumor microenvironment by regulating metastasis, angiogenesis, and immunity [107]. The first study highlighting the role of exosomes in cancer was reported in 1996 by Zitvogel and his colleagues [108]. They reported that exosomes released by DCs could promote the induction of CD8+ T-lymphocyte dependent anti-tumor immune responses in mice *in vivo* (77). Because of these findings, they concluded that exosome-based cell-free vaccines could be used as an alternative method for DC adoptive therapy against tumors [106].

## CONCLUSION

Exosomes are tiny sized vesicles derived from cells that facilitate intercellular communication processes. Numerous studies have demonstrated that virally infected cells alter the host exosomal composition (Table **1**). Many viruses can hijack host exosome pathways. Modified exosomes contribute to viral transmission and immune invasion. This review highlights the role of exosomes as a vehicle/non-classical entry pathway for RNA and DNA viruses.

There have been numerous amounts of studies highlighting the relationship of RNA viruses, immune response and exosome regulation. However, the impact of DNA virus infections and their effect on exosome biogenesis have not yet been completely explained. Exosomes can help the host restrict and/or enhance immune responses against pathogens by activating anti-viral mechanisms. Currently, in our laboratory, we are evaluating exosome biogenesis and composition and their role(s) in DNA viral infection and/or host protection. Clarifying these mechanisms may lead to better diagnostics and alternative therapeutics against a variety of common and emerging viral infections.

## Figures and Tables

**Table 1:** Relationship of Exosomes in RNA and DNA Virus Infection.

**Exosome** **Biogenesis**	**Viral Components Harbored in Exosomes**	**DNA Viruses**	**RNA** **Viruses**	**Roles of Exosomes in Viral Infection**	**Exosome Diagnostic and Therapeutic in Viral Infections**
**Early endosome development during endocytosis** [56, 71]	mRNA miRNA DNA [12]	Ads [36]	Ebola [59]	Attaches cell surface CAR receptor onto host cells [36]	Encourages the release of exosomes encompassing the HIV- 1 genome to free the body of viral factors [81]
**ILVs are released from cells produced by the budding of endosomal MVBs** [14, 16]	Tetraspanins (CD9, CD63, CD 81, CD82) [12]	EBV [98]	HCV [43]	Recruits constituents of the host ESCRT mechanism to the sight of viral maturation *via *Trojan hypothesis [42]	Targets the HIV-1 genome that have been contrived for effective degradation [81]
**ESCRTs are released to the site of budding** [16]	Immunoregulator molecules (MHCI and MCII) [1]	HPV [109]	DENV [1]	Blocks the development of syngeneic tumor cells vaccinated after immunization [56]	Used as vaccines against tuberculosis [49] and Toxoplasmosis [42]
**ESCRT I and II stimulate membrane budding** [16]	Cytoskeletal proteins (Actin, Tubulin, Lamin, Myosin) [1]	Polyoma virus [98]	HIV [62]	Support viral reproduction and pathogenesis by facilitating exhaustion of MHC-1 and CD4 particles when Nef proteins are released [77]	Used as analytic indicators in HIV-1 [37]
**ESCRT III completes budding** [16]	Enzymes (Glyceraldehyde 3-phosphate dehydrogenase (GAPDH), 3-phosphoglycerate kinase (PGK), pyruvate kinase (PK), Enolase, ATPase) [1]	Hepatitis B [110]	HAV [59]	Initiate plasmacytoid dendrite cells(pDCs) to release interferon [102]	Used as a drug delivery tool for systematic or targeted transport to particular tissues or organs [1]
**Exosomes traffic DNA, RNA, lipids, and proteins** [111]	Heat shock proteins (HSP60, HSP70, HSP90) [1]	HSV [4]	DENV [60]	Efficient transport of suppressed membrane protein 1(LMP1) to target cells [12]	Used as immunomodulators to stimulate or suppress the immune system [56]
